# The Risk Factors for Acute Cerebrovascular Accident (Stroke) in Patients with Severe Acute Respiratory Syndrome Coronavirus (SARS-CoV-2)

**DOI:** 10.3390/v15051140

**Published:** 2023-05-10

**Authors:** Badi A. Alotaibi, Jehad A. Aldali, Hamzah J. Aldali, Sultan Ayoub Meo, Glowi A. Alasiri, Emadeldin M. Elsokkary, Naser D. Alotaibi, Faizah Alotaibi

**Affiliations:** 1 Department of Clinical Laboratory Sciences, College of Applied Medical Sciences, King Saud Bin Abdulaziz University for Health Sciences, Riyadh 11481, Saudi Arabia; 2 King Abdullah International Medical Research Center, Riyadh P.O. Box 3660, Saudi Arabia; 3 Department of Pathology, College of Medicine, Imam Mohammad Ibn Saud Islamic University, Riyadh 13317, Saudi Arabia; 4 Cellular and Molecular Medicine, College of Biomedical Science, University of Bristol, Bristol City BS8 1TD, UK; 5 Department of Physiology, College of Medicine, King Saud University, Riyadh 11461, Saudi Arabia; 6 Department of Biochemistry, College of Medicine, Imam Mohammad Ibn Saud Islamic University, Riyadh 13317, Saudi Arabia; 7 Department of Psychology, Imam Mohammed Ibn Saud Islamic University, Riyadh 13317, Saudi Arabia; 8 Neurology Division, King Abdulaziz Medical City, College of Medicine, King Saud Bin Abdulaziz University for Health Sciences, Riyadh 14611, Saudi Arabia; 9 College of Science and Health Professions, King Saud Bin Abdulaziz University for Health Sciences, Alahsa 31982, Saudi Arabia; 10 King Abdullah International Medical Research Center, Ministry of National Guard-Health Affairs, Riyadh 11481, Saudi Arabia

**Keywords:** SARS-CoV-2, COVID-19, ischemic stroke, cardiovascular disease, risk factors

## Abstract

Severe Acute Respiratory Syndrome Coronavirus (SARS-CoV-2) patients may experience an acute ischemic stroke; however, risk factors, in-hospital deaths, and outcomes have not been thoroughly investigated. This study investigates the risk factors, comorbidities, and outcomes in patients with SARS-VoV-2 infection and acute ischemic stroke compared to patients without these conditions. The present retrospective study was conducted in the King Abdullah International Medical Research Centre (KAIMRC), Ministry of National Guard, Health Affairs, Riyadh, Saudi Arabia, during the period from April 2020 to February 2022. This study investigates the risk variables among the individuals who were diagnosed with either SARS-CoV-2 with stroke or patients with stroke alone. A total of 42,688 COVID-19 patients were registered, 187 cases of strokes were listed in COVID-19 patients, however, 5395 cases with stroke without SARS-CoV-2 infection. The results revealed that factors including age, hypertension, deep vein thrombosis, and ischemic heart disease are associated with an increased risk of ischemic stroke. The results also displayed an elevated frequency of in-hospital deaths in COVID-19 patients with acute ischemic stroke. The results also showed that SARS-CoV-2 together predicts the probability of stroke and death in the study sample. The study findings conclude that ischemic strokes were infrequent in patients with SARS-CoV-2 and usually occur in the presence of other risk factors. The risk factors of ischemic strokes in patients with SARS-CoV-2 are old age, male gender, hypertension, hyperlipidaemia, DVT, ischemic heart disease, and diabetes mellitus. Furthermore, the results showed a higher frequency of in-hospital deaths in COVID-19 patients with stroke compared to COVID-19 patients without stroke.

## 1. Introduction

The Severe Acute Respiratory Syndrome Coronavirus 2 (SARS-CoV-2), also known as the COVID-19 pandemic, was confirmed as a new respiratory viral disease that has developed a threat to the global healthcare system [1] and economies. The rapid spread of COVID-19 across the world led to a global pandemic and public health crises by increasing mortality and morbidity rates. According to the World Health Organization (WHO), there are approximately 758.39 million cases and 6.85 million deaths worldwide caused by the COVID-19 pandemic [2]. In the last two decades, the coronavirus family, which is likely to be originated from zoonotic events, has emerged as common human pathogenic viruses. SARS-CoV initially spread in China in 2003 which led to approximately 88,422 cases with 916 deaths in 26 different countries [3]. Similarly, in 2012, another coronavirus, MERS-CoV, spread in Saudi Arabia, causing 2949 cases and 858 deaths [3].

The rate of spread of SARS-CoV-2, however, is greater than both SARS-CoV and MERS-CoV. Coronaviruses contain a spike glycoprotein found on the viral envelope, which binds to a specific host receptor called the angiotensin-converting enzyme 2 (ACE2) receptor to allow viral entry into the host cells [4]. Previously, SARS-CoV was found to enter host cells, which express ACE2, which is produced in several organs such as the lung, kidney, gastrointestinal tracts, and neurological system, including the brain [4]. The spike glycoprotein binds to the subdomain of ACE2, allowing viral fusion into the membrane with the help of some host transmembrane proteins such as transmembrane protease serine 2 (TMPRSS2) and disintegrant metallopeptidase domain 17 (ADAM17) [4,5]. The process stimulates pro-inflammatory cytokines, and chemokines activate the inflammatory response to battle the virus [4]. The cell-free and macrophage-phagocytosed viruses enter the blood circulation and cause the infection of organs with high expression levels of ACE2 [4]. The clinical symptoms of COVID-19 are closely similar to influenza infection [6]. It is mainly characterized by a high temperature in 90% of patients with coughing, myalgia, and fatigue in 50% [7]. Acute myocarditis was shown in 12% of infected patients, and gastroenteritis and diarrhoea in 5% of infected patients [7,8]. Moreover, pneumonia was one of the main complications diagnosed among SARS-CoV-2 patients [9]. Often, mortality rates were high in advanced-age patients with comorbidities including cardiac or respiratory disorders [9]. In addition, clinical characteristics of COVID-19-infected patients showed depressed levels of lymphocytes in the blood, which may lead to death [8,10]. In response to COVID-19, acute inflammation response is marked by the elevation of cytokines such as TNFα, IL6, and IL10 [10].

Globally, researchers are exploring the risk factors that might contribute to COVID-19 disease severity [11]. The World Health Organization (WHO) revealed that elderly individuals and people with underlying medical conditions are at increased risk for developing severe COVID-19 disease, and most COVID-19 deaths are linked to the existence of comorbidities [11]. The epidemiological and clinical features of patients with COVID-19 have been investigated by several researchers. However, the risk factors and mortality have not been sufficiently explored [12]. Most comorbidities that have been recognised as high-risk factors are diabetes mellitus, hypertension, stroke, cancer, and kidney issues [11]. Furthermore, neurological patients were found to be affected by SARS-CoV [13]. Similarly, neurological disorders could be related to cardiorespiratory failure and metabolic disorders induced by COVID-19 infection [9]. 

A study in Wuhan city, China, showed that COVID-19 was significantly related to neurological disorders such as stroke, impaired levels of consciousness, and muscle damage [14]. In addition, a study of SARS-CoV in Taiwan showed that three patients had axonal polyneuropathy, two exploited myopathies, and five showed large vessel ischemic stroke [13]. Furthermore, it was previously shown that SARS-CoV causes brain infection in transgenic mice by binding to ACE2 [15]. It was then further investigated to show that the virus entered the brain through the olfactory bulb. The death of mice was caused by the failure of cardiorespiratory centres in the medulla and minimal cellular infiltration in the brain [15]. The interaction between COVID-19 and ACE2 receptor leads to depressed levels of ACE2, which leads to hyper-inflammation, vasoconstriction, coagulation, and hypertension and increases the risk of ischemic strokes.

Moreover, several disorders may develop as consequence of COVID-19, including thrombophilia and disseminated intravascular coagulation [16]. Furthermore, COVID-19 may induce myocarditis which leads to stroke [17]. In addition, brain endothelial cells have ACE2 receptors, which may cause arterial and venous cerebrovascular disease due to blood vessel inflammation [9]. It is also worth noting that diabetes mellitus was found to be one of the serious comorbidities that is associated with the severity of COVID-19 infection. Diabetic patients have an elevated risk for severe side effects involving multi-organ failure and adult respiratory distress syndrome [11]. Approximately 20–50% of patients with COVID-19 had diabetes, which stresses the significant importance of the association between diabetes and COVID-19 [18]. During the COVID-19 pandemic, a higher morbidity rate was found in diabetic patients than in non-diabetic patients and was highly implicated in acute ischemic stroke [19,20]. The several studies have linked diabetes mellitus with cerebral stroke in incidence and poor outcomes [21,22]. It has also been reported that old age, dyslipidemia, hypertension, coronary heart disease, and smoking are recognized risk factors for ischemic stroke [23]. In the Middle East region, patients with acute ischemic stroke have a high comorbidity burden compared to patients from developed countries [24]. In Saudi Arabia, the annual incidence of stroke is 0.029% (95% CI: 0.015 to 0.047), equivalent to 29 strokes per 100,000 people annually [25]. In this current study, we investigate the differences in demographics, clinical characteristics, in-hospital events, and outcomes between COVID-19 patients who had an ischemic stroke, COVID-19 patients who did not have a stroke, and stroke patients to further elucidate the incidence of stroke.

## 2. Subjects and Methods

### 2.1. Study Design and Settings

The present retrospective study was conducted in the King Abdullah International Medical Research Centre (KAIMRC), Ministry of National Guard, Health Affairs, Riyadh, Saudi Arabia, during the period from April 2020 to February 2022.

### 2.2. Inclusion and Exclusion Criteria

The inclusion criteria were patients diagnosed with either COVID-19, COVID-19 with cerebrovascular accident, stroke, or stoke alone and Saudi and non-Saudi patients of all age groups. However, data records that did not include any of the above information were excluded from the study.

### 2.3. Data Collection

After the ethical approval, for the purpose of data collection, the research team members contacted the medical records unit, department of research and data management section at the King Abdullah International Medical Research Centre (KAIMRC) in the Ministry of National Guard-Health Affairs, Riyadh, Saudi Arabia. The data were retrieved from the electronic health records of the patients through the hospital data management systems. During the study period, a total of 42,688 COVID-19 patients were enrolled, and 187 patients had ischemic strokes. There were a total of 5395 hospital admissions due to strokes that did not involve COVID-19.

The research team investigated the demographics, clinical characteristics, in-hospital deaths, and outcomes between COVID-19 patients who had an ischemic stroke (Group 1), COVID-19 patients who did not have a stroke (Group 2), and stroke patients (Group 3). 

The clinical data including demographic information and risk factors such as deep vein thrombosis (DVT), ischemic heart disease, pneumonia, hyperlipidaemia, diabetes mellitus, and hypertension, as well as in-hospital deaths and patient outcomes were recorded and included in the Excel sheet. 

### 2.4. Statistical Analysis

A comparison of patients’ age, gender, risk factors, and discharge from the hospital status among COVID-19 patients was conducted based on the presence or absence of stroke. The data were collected and analysed using the chi-squared test (χ^2^ test) for categorical data and the one-way ANOVA test for continuous data to identify significant differences between COVID-19 patients with and without stroke. The binary logistic regression analysis was employed for COVID-19 stroke predictors and for death predictors, and a *p* value (*p* < 0.05) is considered significant.

## 3. Results 

### 3.1. Comparison between COVID-19 Patients with and without Stroke and Stroke Patients

A total of 42,688 COVID-19 patients were enrolled, and 187 strokes were registered in COVID-19 patients. There was a total of 5395 hospital admissions due to strokes that did not involve COVID-19. The mean age (years ± standard deviation) of COVID-19 patients without any stroke was significantly lower than patients with stroke and COVID-19 and stroke patients, respectively (36.99 ± 18.27 vs. 63.52 ± 11.96 vs. 62.28 ± 13.14: *p* < 0.001) (Table 1). Patients with acute stroke and COVID-19 had the highest average age, while the patients with COVID-19 but without stroke and patients with acute stroke but no COVID-19 had the lowest average ages (Table 1). Men were more likely to have COVID-19 and stroke compared to females. Of note, there were unspecified genders among the data, which represents 0.01% COVID-19 patients without any stroke (Table 1). 

Moreover, the proportion of hypertension was significantly higher in COVID-19 patients with stroke compared to COVID-19 patients without stroke or patients with stroke only (126 (67.38% vs. 1959 (4.59%) (Table 1). Similarly, DVT cases were significantly higher in COVID-19 patients with stroke compared to COVID-19 patients without stroke or patients with stroke only (7 (3.74% vs. 82 (0.19%) vs. 88 (1.63%). Likewise, for ischemic heart disease, pneumonia, hyperlipidemia, and diabetes, results indicate that there are statistically significant differences between the three groups in the preceding variables, as seen in Table 1.

### 3.2. Outcomes Comparison between COVID-19 Patients with and without Stroke and Stroke Patients 

The results showed that there are statistically significant differences between the three study groups in terms of patients’ outcomes and in-hospital deaths. The results comparing the three groups are represented in (Table 2): patients with acute stroke and COVID-19, patients with COVID-19 but no stroke, and patients with acute stroke and no COVID-19. In the discharge home and in-hospital death, the significance levels were statistically significant where the *p* value was < 0.01, indicating that there are substantial variations between the three groups in the two outcomes. The proportion of COVID-19 patients who were discharged home without stroke was significantly higher than other groups (99.13% vs. 98.40% vs. 98.20%: *p* < 0.001). With regards to hospital deaths, there was a significant decrease in the number of deaths in COVID-19 patients with stroke compared to other groups (1.60% vs. 0.87% vs. 1.80% *p* < 0.001) (Table 2). 

### 3.3. Death and Stroke Predictors 

The binary logistic regression model was performed in order to predict stroke and death. The results showed that the model is statistically significant, as indicated by the level of significance of the Omnibus Tests of Model Coefficients test (*p* < 0.001). This indicates that the two variables (stroke and COVID-19) together predict a way that is statistically indicative of the probability of death in the study sample, whereas neither variable predicts the probability of death individually (*p* > 0.05) (Table 3). 

Similarly, the model is significant as shown by the level of significance of the Omnibus Tests of Model Coefficients test (*p* < 0.001), meaning that the variables hypertension, DVT diagnosis, ischemic heart disease, pneumonia, hyperlipidemia, diabetes mellitus, and COVID-19 predict the probability of stroke in a statistically significant manner, and the levels of significance are statistically significant (Table 4).

## 4. Discussion

The recent literature has linked COVID-19 with increased risk factors of ischemic stroke [22]. COVID-19 may raise the risk of acute ischemic stroke, which is comparable to the increased risk of 3.2-fold to 7.8-fold that is reported during the first 3 days following other respiratory tract infections [22]. The aim of this study is to investigate the risk variables and significant differences in demographics, clinical characteristics, in-hospital deaths, and outcomes between COVID-19 patients with stroke, COVID-19 patients without stroke, and stroke patients. We investigated different variables such as age, gender, cardiovascular risk factors, and discharge status of COVID-19 patients based on the presence or absence of stroke. The results revealed that patients with COVID-19 and stroke tend to be old in age, more likely to be men, and have an increased percentage of hypertension, DVT, ischemic heart disease, hyperlipidaemia, and diabetes mellitus. However, pneumonia in COVID-19 patients with stroke represents a reduced frequency (5.35%) compared to stroke patients (10.55%) (Table 1). These results indicate that ageing in COVID-19 patients with stroke may affect the patient’s condition as well as having increased risk for cardiovascular diseases such as hypertension, DVT diagnosis, ischemic heart disease, hyperlipidaemia, and diabetes. 

Moreover, our findings showed that there is an increased percentage of COVID-19 patients without stroke in terms of home discharge compared to other groups. In addition, our results also displayed an elevated frequency of in-hospital deaths in COVID-19 patients with stroke compared to those without stroke (Table 3). Overall, these results point to an elevated frequency of death in COVID-19 patients with stroke along with substantial variations between the three groups in the two preceding variables. We used binary logistic regression analysis to predict the probability of death and stroke. Our findings uncover that both stroke and COVID-19 together indicate death probability (Table 4). Moreover, the variables hypertension, DVT diagnosis, ischemic heart disease, pneumonia, hyperlipidemia, diabetes, and COVID-19 might be indicative for stroke probability (Table 4).

Furthermore, researchers noted that patients aged 65 to 74 years old appeared to be at higher risk of stroke from COVID-19 than older patients and people without a history of stroke [26]. However, more studies are required to explore stroke subtypes incidence by age group. This might be attributable to a number of age-related pathologies and processes, such as cerebral microembolism, white matter lesions, thickening of the vascular basement membrane, and increased blood brain barrier permeability, which result in endothelial damage, altered vessel elasticity, and erratic blood flow and pressure, which impair autoregulation and raise the risk of intracerebral haemorrhage [27].

ACE2 shows an elevated level of expression in SARS-CoV-2-infected patients as it is required by COVID-19 to enter the host cells. ACE2 is considered as the binding site for the virus glycoprotein spike, which marks it as a therapeutic target for COVID-19. It has been shown that ACE2 expression is encoded by X chromosome, suggesting that its regulation might differ between males and females. Previously, it was demonstrated that ACE2 expression in the lung is lower in women, suggesting that estrogen might suppress ACE2 expression. In addition, mucosa-specific serine protease TMPRSS2, which facilitates virus entry into human host cells, is upregulated by androgen. This further suggests that sex hormones might facilitate the infection of males by COVID-19 [28,29]. In addition, the severity of COVID-19 infection and complications in males were found to be more compared to females, especially in complications such as acute respiratory distress syndrome, secondary infection, acute cardiac injury, coagulopathy, acute kidney injury, and arrhythmia [28]. In our study, stroke together with COVID-19 was demonstrated in males more than females. This suggests that stroke incidence with COVID-19 could be affected by gender. Moreover, our results revealed an increased frequency of cardiovascular disease risk factors such as hypertension, DVT diagnosis, ischemic heart disease, hyperlipidaemia, and diabetes. Patients with COVID-19 may also suffer significant changes in blood pressure (BP), which makes them more vulnerable to haemorrhagic stroke events because a lower ACE2 expression suggests higher Ang II availability [27]. 

Study strengths and limitations: The strength of this research work is that this study investigates the risk variables between COVID-19 patients with ischemic stroke, COVID-19 patients without stroke, and stroke patients alone. Moreover, such studies are in need of time while establishing the policies and guidelines about COVID-19 and ischemic risk variables. However, due to the retrospective, observational design of this study, we assessed the relationships, not causality. Furthermore, due to the lack of post-discharge information from the COVID-19 cohort, we were unable to report on the long-term results of this population.

## 5. Conclusions

The study findings conclude that ischemic stokes are infrequent in patients with SARS-CoV-2 and usually occur in the presence of other risk factors. The risk factors of ischemic strokes in patients with SARS-CoV-2 are old age, male gender, hypertension, hyperlipidaemia, DVT, ischemic heart disease, and diabetes mellitus. Furthermore, the results showed a higher frequency of in-hospital deaths in COVID-19 patients with stroke compared to COVID-19 patients without stroke.

## Figures and Tables

**Table 1:** Demographical and clinical characteristics of the study participants.

Characteristics	Patients with Acute Stroke and COVID-19 *n* = 187	Patients with COVID-19 without Stroke (*n* = 42,688)	Acute Stroke Patients Lacking COVID-19 *n* = 5395	*p* Value
Age < 25	1 (0.53%)	11,129 (26.07%)	83 (1.54%)	<0.001
Age 25–44	14 (7.49%)	18,007 (42.18%)	428 (7.93%)	<0.001
Age 45–69	107 (57.22%)	11,093 (25.99%)	3108 (57.61%)	<0.001
Age ≥ 70	65 (34.76%)	2459 (5.76%)	1776 (32.92%)	0.006
Mean age (±SD)	63.52 ± 11.96	36.99 ± 18.27	62.28 ± 13.14	<0.001
Men	104 (55.61%)	20,556 (48.15%)	3284 (60.87%)	<0.001
Female	83 (44.36%)	22,130 (51.84%)	2111 (39.13%)	<0.001
Hypertension	126 (67.38%)	1959 (4.59%)	1589 (29.45%)	<0.001
DVT	7 (3.74%)	82 (0.19%)	88 (1.63%)	<0.001
IHD	17 (9.09%)	388 (0.91%)	254 (4.71%)	<0.001
Pneumonia	10 (5.35%)	1210 (2.83%)	569 (10.55%)	<0.001
Hyperlipidemia	20 (10.70%)	407 (0.95%)	80 (1.48%)	<0.001
Diabetes	134 (71.66)	757 (1.77%)	1592 (29.51%)	<0.001

IHD: ischemic heart disease; DVT: deep vein thrombosis.

**Table 2:** Patients Outcomes.

Outcome	Patients with Acute Stroke and COVID-19 *n* = 187	Patients with COVID-19 without Stroke (*n* = 42,688)	Acute Stroke Patients Lacking COVID-19 *n* = 5395	*p* Value *
Discharge home	184 (98.40%)	42,317 (99.13%)	5298 (98.20%)	<0.001
In-hospital death	3 (1.60%)	371 (0.87%)	97 (1.80%)	<0.001

* The data were collected and analysed using the chi-squared test (χ^2^ test).

**Table 3:** Binary logistic regression analysis for death predictors.

Predictor	B	S.E.	Wald’s χ^2^	df	*p* Value	OR (Odds Ratio)	95% C.I. for EXP(B)	
							Lower	Upper
Constant	−4.116	0.582	50.017	1	<0.001	0.016	-	-
Stroke (1 = Yes, 0 = No)	−0.620	0.584	1.127	1	0.288	0.538	0.171	1.690
COVID-19 (1 = Yes, 0 = No)	0.116	0.591	0.038	1	0.844	1.123	0.353	0.3.576
**Test**			**χ^2^**	**df**	***p*** **Value**			
Omnibus Tests of Model Coefficients			36.083	2	<0.001			
Variable(s) entered: Stroke, COVID-19. −2 Log likelihood = 5262.49; Cox and Snell R^2^ = 0.001; Nagelkerke R^2^ = 0.007								

**Table 4:** Binary Logistic Regression Analysis for Stroke Predictors.

Predictor	B	S.E.	Wald’s χ^2^	df	*p* Value	OR (Odds Ratio)	95% C.I. for EXP(B)	
							Lower	Upper
Constant	20.720	500.266	0.002	1	0.967	996,769,911.4	-	-
Hypertension (1 = Yes, 0 = No)	1.995	0.201	98.407	1	<0.001	7.349	4.955	10.898
DVT Diagnosis (1 = Yes, 0 = No)	1.725	0.559	9.514	1	0.002	5.613	1.876	16.799
Ischemic Heart Disease (1 = Yes, 0 = No)	0.465	0.317	2.149	1	0.143	1.592	0.855	2.966
Pneumonia (1 = Yes, 0 = No)	−1.353	0.353	14.715	1	<0.001	0.259	0.130	0.516
Hyperlipidemia (1 = Yes, 0 = No)	0.890	0.311	8.195	1	0.004	2.434	1.324	4.477
Diabetes (1 = Yes, 0 = No)	3.903	0.205	361.314	1	<0.001	49.539	33.127	74.083
COVID-19 (1 = Yes, 0 = No)	−27.631	500.266	0.003	1	0.956	0.000	0.000	0.000
**Test**			**χ^2^**	**df**	***p*** **Value**			
Omnibus Tests of Model Coefficients			33163.06	7	<0.001			
Variable(s) entered: Hypertension, DVT Diagnosis, Ischemic Heart Disease, Pneumonia, Hyperlipidemia, Diabetes, COVID-19. −2 Log likelihood = 1412.69; Cox and Snell R^2^ = 0.479; Nagelkerke R^2^ = 0.972								

## Data Availability

May be provided on reasonable request to the corresponding author.
